# Simplified setup for the vibration study of plates with simply-supported boundary conditions

**DOI:** 10.1016/j.mex.2019.09.023

**Published:** 2019-09-19

**Authors:** Patrick Dumond, Dominic Monette, Fadi Alladkani, James Akl, Inès Chikhaoui

**Affiliations:** University of Ottawa, Canada

**Keywords:** Non-destructive and reusable simply-supported plate vibration test method, Experimental measurements, Flexible test rig, Reusable specimens, Simply supported boundary conditions, Kirchhoff plates, Ribbed plates

## Abstract

The experimental study of vibrating plates having simply-supported boundary conditions can be difficult to achieve due to the complexity of preventing translation, but allowing rotation along all boundaries simultaneously. Only a few methods have been proposed, but all are either time-consuming to set up and involve customization of the test rig for each plate or do not allow the plate to be reused for other purposes. The method described in this paper offers a low-cost, simple, accurate and non-destructive way of experimentally measuring the modal properties of thin, simply supported plates and can be used for quick validations of models and designs without modification for multiple trials and varying plate properties. The key attributes of this method include:

• An adjustable sliding support frame which can be made of a distinct material from the plate and which can accommodate variations in plate geometry and properties without modification.
• Removable flexible sealant applied in a v-groove on the supporting frame which can be easily used to fix and support the plate according to the simply-supported boundary conditions.
• A low-profile design, which can be used to accommodate most experimental testing methods for determining modal properties of vibrating plates.

## Graphical abstract

**Figure:**
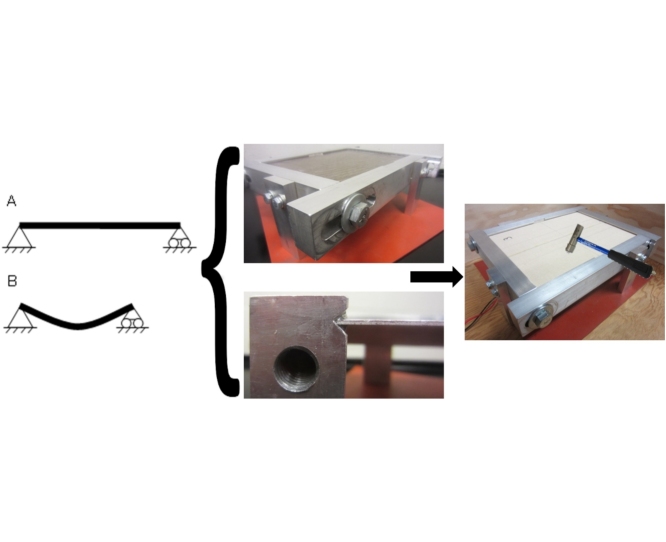

**Specification Table**

**Table:** 

Subject Area:	Engineering
More specific subject area:	Mechanical Vibration
Method name:	Non-destructive and reusable simply-supported plate vibration test method
Name and reference of original method:	See Additional Information section of the paper
Resource availability:	N/A

## Method details

### Materials

**Fig. 1:**
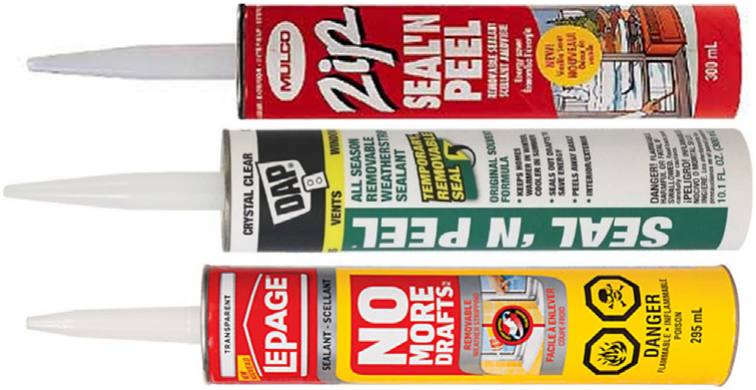
Removable weather stripping sealants.

• Aluminum or steel bar stock for the supporting frame.
• Bolts and washers to assemble the supporting frame.
• Rubber, foam or silicone vibration isolation pad to place under the supporting frame.
• A flat and level surface on which to rest the supporting frame during plate installations and experiments.
• Removable weather stripping sealant (e.g. Mulco/DAP Seal ‘N Peel or LePage No More Drafts, Fig. 1).

### Frame fabrication

**Fig. 2:**
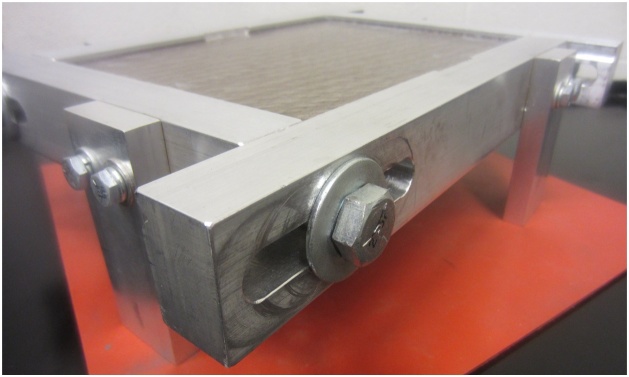
Sliding slots to accommodate plates of varying sizes.

**Fig. 3:**
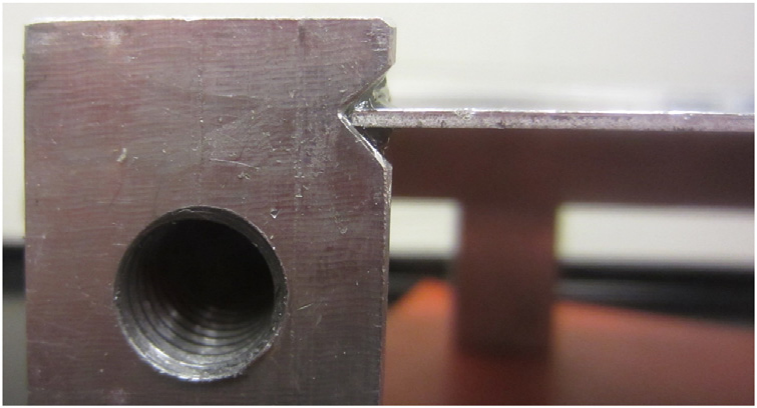
V-groove supporting the plate with removable sealant beads above and below.

**Fig. 4:**
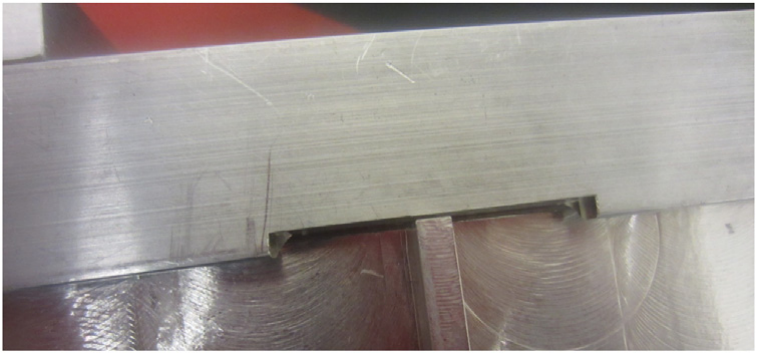
Cutout to accommodate surface mounted elements.

**Fig. 5:**
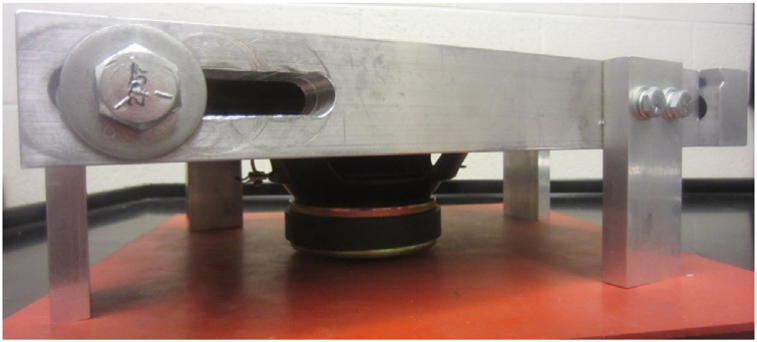
Supporting frame legs to provide airflow and accommodate vibration generators or speakers.

**Fig. 6:**
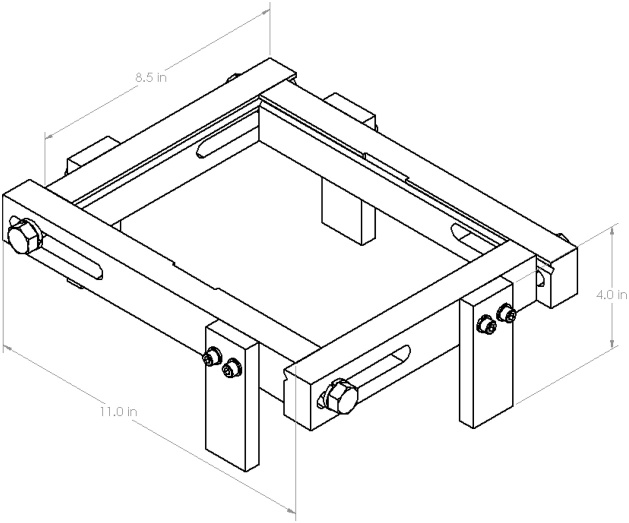
Sample frame design.

The frame can be designed to accommodate plates of various sizes. Important features include: sliding slots to accommodate minor variations in plate dimensions (Fig. 2), a v-groove to support the plate and limit edge displacements, but which allows edge rotation along its length and width (Fig. 3), cutouts to accommodate elements which are surface mounted on the plate, if required (Fig. 4), and legs that allow air flow below the plate and which can accommodate vibration generators or speakers, if required (Fig. 5). A sample frame design is shown in Fig. 6.

### Setup

**Fig. 7:**
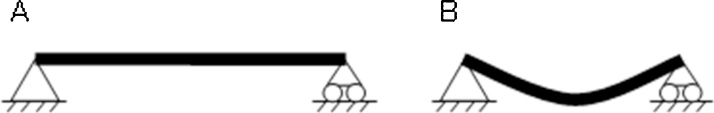
(A) flat stationary plate, (B) bent plate due to vibration.

Simply supported boundary conditions require supports which are flexible enough to allow in-plane shortening during bending, but rigid enough to prevent lateral displacement during rotation (Fig. 7).

**Fig. 8:**
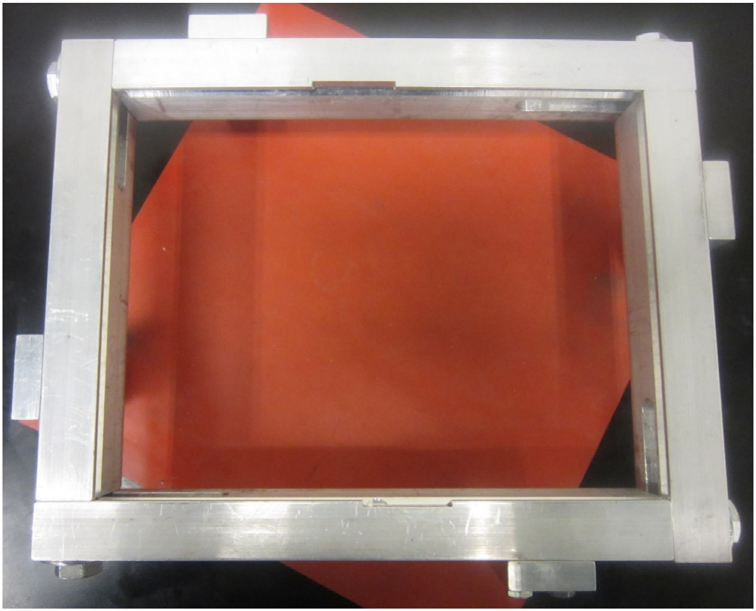
Frame extended to its maximum size.
2 Apply sealant in the middle of each v-groove (Fig. 9). Enough sealant is applied if sealant is visible on all edges of the plate, both above and below (Fig. 3). Too much sealant is applied if sealant begins to extend onto the plate surface.

Flexible weather stripping sealant has been found to provide the required stiffness for this purpose and reduces vibration transmission to the supporting frame. Removable sealants, as shown in Fig. 1, are also easy to remove between tests and do not damage the plate being tested. Care must be taken when using plates made of materials that are sensitive to or can be damaged by solvents, as most removable sealants dry via solvent evaporation. Using a sliding frame, such as the one shown in Fig. 2, accommodates minor or major differences in plate dimensions during installation of the plate. The use of a vibration isolating pad between the supporting frame and the table has also been found to help reduce signal noise. Installation procedure:

1 Loosen frame support bolts and slide frame to its maximum opening size (Fig. 8).

**Fig. 9:**
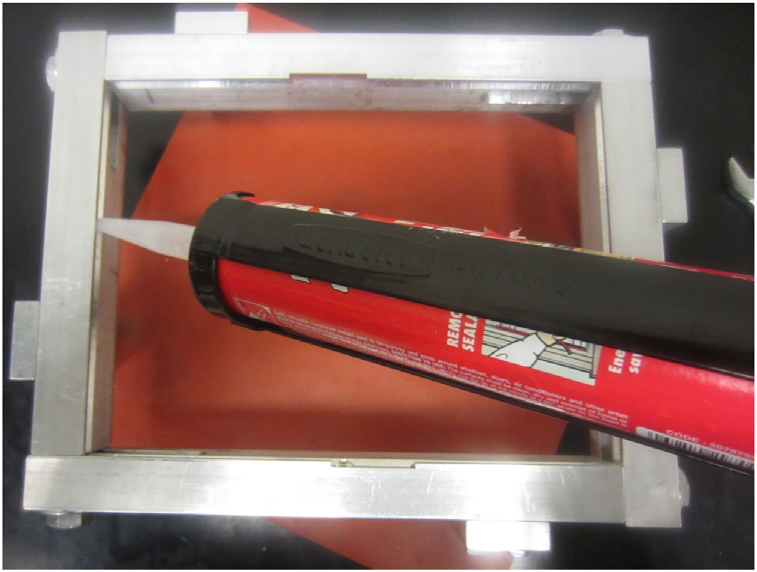
Application of sealant in the v-groove.
3 Align plate with v-grooves in the middle of the opening. If attachments are included, align attachments with supporting frame cutouts.
4 Slide two parallel frame supports until they make contact with the plate (Fig. 10), then slide the opposing two frame supports until they also make contact with the plate (Fig. 11). Apply light finger pressure on all sides to ensure proper plate contact with the frame. Too much pressure may induce unwanted compressive stresses in the plate or even cause bending in thinner plates.

**Fig. 10:**
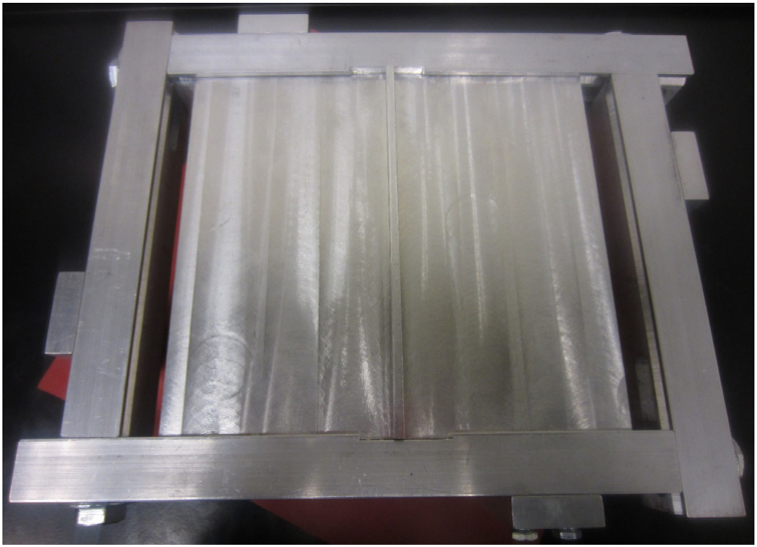
First two edges of frame in contact with the plate.

**Fig. 11:**
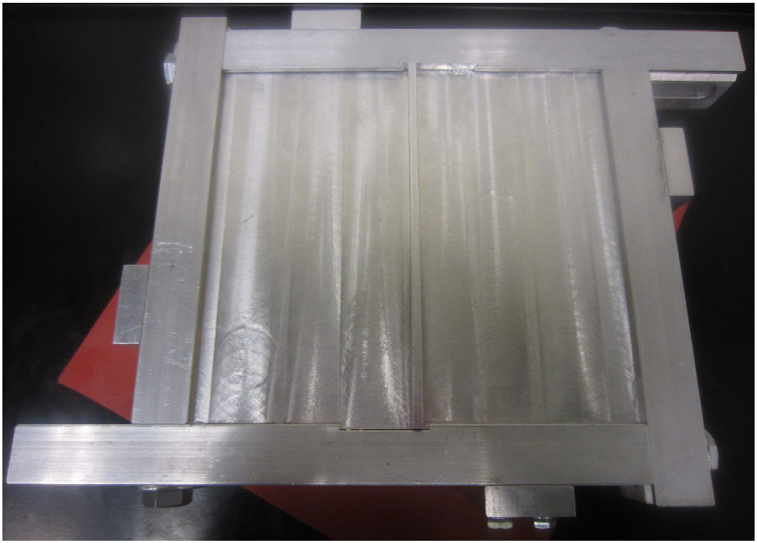
Fully supported plate.
5 Tighten bolts to approximately 9 Nm torque, while ensuring the frame remains perfectly flat. Tightening should occur progressively by tightening each bolt in a diagonal pattern to ensure even pressure on the plate. Overtightening of the bolts tends to rotate and misalign the frame elements, causing the plate to sit poorly in the v-grooves. 9 Nm has been found to hold the frame together snuggly during testing without loosening. Verify flatness of the frame after tightening.
6 Tap the plate lightly with your finger to ensure that enough sealant has been applied and that no direct contact is made between the plate and the frame.

The setup can now be used to test the modal properties of simply supported plates using a variety of excitation techniques, such as a speaker, shaker or impact hammer, in combination with a visualization and/or measurement method, such as Chladni patterns, accelerometers or a scanning laser vibrometer. Metal, polymer and composite plates have been tested successfully using this method. However, the recommended setup might need to be modified to account for other plate materials. Two simple testing methods are described below.

### Procedure

Any number of experimental modal analysis techniques can be used with the setup described above. Two relatively low-cost techniques that have been used successfully are detailed below.

Testing Method 1: Speaker and Chladni Pattern Method

**Fig. 12:**
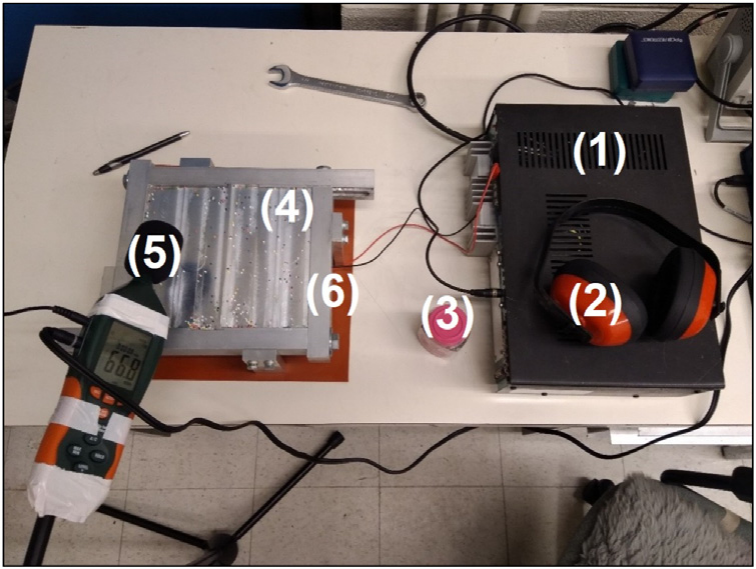
Experimental setup for the speaker/Chladni pattern testing method.

A speaker is placed at 3 mm below the underside of the plate, measured from the top of its peripheral, and is set to emit sound at about 40 W. Finely divided material, such as cake sprinkles, are sprinkled on the plate. Computer-generated sine waves are amplified and emitted below the plate, which vibrates and displaces the sprinkles forming distinct patterns (mode-shapes) along vibration nodal lines at resonant frequencies of the plate. A receiver, such as a sound level meter, measures acoustic sound intensity while sine wave frequencies are swept. A natural frequency is reached when the receiver displays a local maximum value and when the sprinkles assume a Chladni pattern. Practically, this method has been found to be limited to about 1200 Hz for such a 50 W nominal speaker. The frame shown in Fig. 6 is also limited to using a 50 W speaker, as this is the smallest speaker diameter that fits below this frame. In theory, higher frequency measurements could be achieved on a larger plate with a more powerful speaker. However, proper sound insulation would be required as frequencies above 1200 Hz are uncomfortable for the operator, even while wearing ear protection. The speaker and Chladni pattern method setup is described in Fig. 12 and Table 1.

**Table 1:** Items used in the experimental setup for the speaker/Chladni pattern testing method.

Item	Specifications
(1) Signal Amplifier	BOGEN Classic C60 Amplifier - 60 W RMS.
(2) Acoustic Earmuffs	Protects the subject from the loud generated sounds.
(3) Cake Sprinkles	Moves along with the mode-shapes being light and non-adhesive.
(4) Plate and Frame	Joined using a removable sealant and fastened with a wrench.
(5) Sound Receiver	EXTECH HD600 Datalogging Sound Level Meter.
(6) Sound Emitter	Diameter 5.5", nominal 50 W, maximum 120 W, impedance 8 Ω.

Testing method 2: Impulse Hammer and Accelerometer Method

**Fig. 13:**
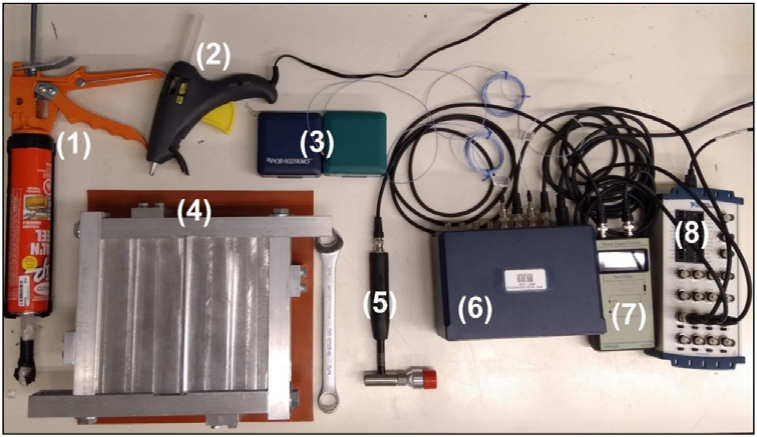
Experimental setup for the impulse hammer/accelerometer testing method.

Accelerometers are glued to the surface of the plate using hot glue and the plate surface is impacted using an impulse hammer. Appropriate locations for placing accelerometers and impacting the plate when measuring the six lowest modes of vibration are shown in Fig. 16. These locations should be chosen to be simultaneously as close to as many antinodes under consideration as possible, while avoiding nodes completely. Both the impact force using a load cell and the response using accelerometers are measured. The signals are then processed by a signal conditioner and captured by a data acquisition (DAQ) device connected to a computer for further processing and modal extraction. When impacting the plate, ensure the hammer remains orthogonal to the plate and restrict the force to a range 25 to 65 N for plates that fit within the frame shown in Fig. 6. This range has been found to provide optimal signal to noise ratios for extracting modal information. However, an appropriate impact force range will need to be determined for plates of other dimensions. Care must be taken not to impact the plate with such a large force that its resulting deformation would cause the plate to separate from the removable sealant. Signal processing should be considered carefully to ensure accurate results. Accelerometer and impulse hammer signals should be isolated. Logarithmic decrements for modal analysis can be used to convert impulses and accelerations into natural frequencies. The Least-Squares Complex Exponential (LSCE) algorithm was found to be best suited for this experiment. Finally, the window size (range of data points recorded on impact) must be optimized to obtain the cleanest results. The setup is described in Fig. 13 and Table 2.

**Table 2:** Items used in the experimental setup for the impulse hammer/accelerometer testing method.

Item	Specifications
(1) Sealant Gun and Sealant	Reduces plate-frame contact and minimizes clamping effects.
(2) Glue Gun	Fixes the accelerometers on the plate surface.
(3) Accelerometers	PCB PIEZOTRONICS INC. Model 352C22.
(4) Plate and Frame	Simply-supports the plate and fastened with a wrench.
(5) Impact Hammer	KISTLER Model 9928 – Provides impacts and measures the force.
(6) Signal Conditioner	PCB PIEZOTRONICS INC. Model 482C Series.
(7) Power Supply/Coupler	KISTLER Type 5114 – Powers hammer and outputs signal.
(8) Multifunction I/O Device	NI USB-6212 – DAQ outputs to computer for processing.

## Method validation

**Fig. 14:**
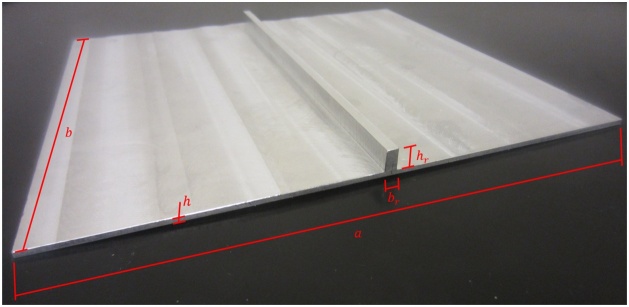
Ribbed plate.

6061-T6 aluminum plates were used to validate the method. Seven plates were tested and two configurations were used: simple flat rectangular plates and a ribbed plate where the rectangular cross-section rib is centered along the plate’s length, as shown in Fig. 14. The ribbed plate was manufactured as one piece.

Material properties of these aluminum plates are given in Table 3.

**Table 3:** Material properties of 6061-T6 aluminum [1].

Parameter		Values
E	Young’s modulus (Pa)	$6.89\cdot 10^{10}$
ρ	Volumetric mass density ($\text{k}\text{g}\;{\text{m}}^{-3}$)	$2.70\cdot 10^{-3}$
ν	Poisson’s ratio	$3.30\cdot 10^{-1}$

Plate configurations and their dimensions used to validate the method are provided in Table 4.

**Table 4:** Plate configurations and dimensions.

Parameter		Plate 1	Plate 2	Plate 3	Plate 4	Plate 5	Plate 6	Ribbed plate
a	Plate length *a* (mm)	246.7	246.6	246.6	246.5	245.5	246.5	206
b	Plate width *b* (mm)	186.1	186.1	186.3	186.2	186.2	186.2	186
h	Plate thickness *h* (mm)	0.81	0.81	0.81	1.23	1.23	1.23	2.15
$b_{r}$	Rib width *b_r_* (mm)	-	-	-	-	-	-	4.36
$h_{r}$	Rib thickness *h_r_* (mm)	-	-	-	-	-	-	7.40

Tested plates range in mass between 0.10-0.24 kg. The supporting frame, as shown in Fig. 6, has a mass of approximately 3.2 kg. This provides a plate to frame mass ratio range of 0.03-0.08, helping to minimize effects of the frame on the plate’s natural frequencies and well below the value determined to be adequate by Robin et al. [2].

To validate the method, the measured natural frequencies of the simply supported flat rectangular aluminum plates were compared to theoretical values provided by the Kirchhoff-Love plate theory [3]:

(1) $$\omega_{m_{x},m_{y}}=\;\pi^{2}\sqrt{\frac{D}{\rho }}\left[{\left(\frac{m_{x}}{L_{x}}\right)}^{2}+{\left(\frac{m_{y}}{L_{y}}\right)}^{2}\right]$$

where *m* is the mode number in each direction, *ρ* is the density of the plate, *L* is the length of the plate along each axis and *D* is:

(2) $$D=\frac{E\cdot h^{3}}{12\left(1-\nu^{2}\right)}$$

In this case, *E* is the modulus of elasticity, *h* is the thickness of the plate and *ν* is Poisson’s ratio. Kirchhoff-Love plate theory is considered to be valid because h/b≪0.1 in all cases [4]. Mindlin plate theory was also considered [5], but provided insignificant improvements on the results for the dimensions listed in Table 4. Results of Eq. (1), along with both experimental methods described above for each simple flat plate using material properties of Table 3 and dimensions of Table 4 are tabulated in Table 5. In this case, Plate 1,2…6 = theoretical frequencies for each mode calculated using Eq. (1), Sp/Ch = Speaker/Chladni pattern testing method and Im/Ac = Impulse hammer/Accelerometer testing method (using the average of two accelerometers placed on the plate, as in Fig. 16).

**Table 5:** Theoretical and experimental natural frequencies of simply supported flat rectangular plates.

Vibration mode	Plate 1 (Hz)	Sp/Ch (Hz)	Error |%|	Im/Ac (Hz)	Error |%|	Plate 2 (Hz)	Sp/Ch (Hz)	Error |%|	Im/Ac (Hz)	Error |%|
$m_{x}=1,\;m_{y}=1$	89.0	92.4	3.8	90.4	1.5	89.0	93.1	4.6	95.1	6.8
$m_{x}=2,\;m_{y}=1$	186.0	195.4	5.1	174.9	5.9	186.0	195.2	4.9	189.4	1.8
$m_{x}=1,\;m_{y}=2$	259.2	263.4	1.6	254.6	1.8	259.2	266.8	2.9	266.5	2.8
$m_{x}=2,\;m_{y}=2$	356.2	359.4	0.9	342	4.0	356.2	364.5	2.3	361.5	1.5
$m_{x}=3,\;m_{y}=1$	347.5	350.6	0.9	333.6	4.0	347.6	355.4	2.3	350.5	0.8
$m_{x}=1,\;m_{y}=3$	542.9	542.7	0.0	540.8	0.4	542.9	552.1	1.7	521.5	3.9

Vibration mode	Plate 3 (Hz)	Sp/Ch (Hz)	Error |%|	Im/Ac (Hz)	Error |%|	Plate 4 (Hz)	Sp/Ch (Hz)	Error |%|	Im/Ac (Hz)	Error |%|
$m_{x}=1,\;m_{y}=1$	89.0	95	6.8	92	3.4	135.2	141.4	4.6	139.8	3.4
$m_{x}=2,\;m_{y}=1$	186.0	197	5.9	186.7	0.4	282.6	287.5	1.7	284.4	0.6
$m_{x}=1,\;m_{y}=2$	259.0	271	4.6	263.8	1.9	393.5	396.4	0.7	392.6	0.2
$m_{x}=2,\;m_{y}=2$	356.0	367.1	3.1	356.4	0.1	540.9	548.5	1.4	541	0.0
$m_{x}=3,\;m_{y}=1$	347.6	357.7	2.9	343	1.3	528.1	532.8	0.9	522.5	1.1
$m_{x}=1,\;m_{y}=3$	542.3	556.3	2.6	516.4	4.8	824.0	823.5	0.1	812.3	1.4

Vibration mode	Plate 5 (Hz)	Sp/Ch (Hz)	Error |%|	Im/Ac (Hz)	Error |%|	Plate 6 (Hz)	Sp/Ch (Hz)	Error |%|	Im/Ac (Hz)	Error |%|
$m_{x}=1,\;m_{y}=1$	135.6	139.2	2.6	138.6	2.2	135.2	139.1	2.9	138.1	2.1
$m_{x}=2,\;m_{y}=1$	284.2	287.2	1.1	284.2	0.0	282.5	286.5	1.4	283	0.2
$m_{x}=1,\;m_{y}=2$	393.9	399.3	1.4	396.4	0.4	393.4	397.7	1.1	389.3	1.0
$m_{x}=2,\;m_{y}=2$	542.5	549.4	1.3	551.8	1.7	540.8	546	1.0	526.1	2.7
$m_{x}=3,\;m_{y}=1$	531.8	533.2	0.3	530.3	0.3	528.1	534	1.1	520.8	1.4
$m_{x}=1,\;m_{y}=3$	824.3	831.3	0.8	838.8	1.8	823.8	820.2	0.4	739	10.3

**Fig. 15:**
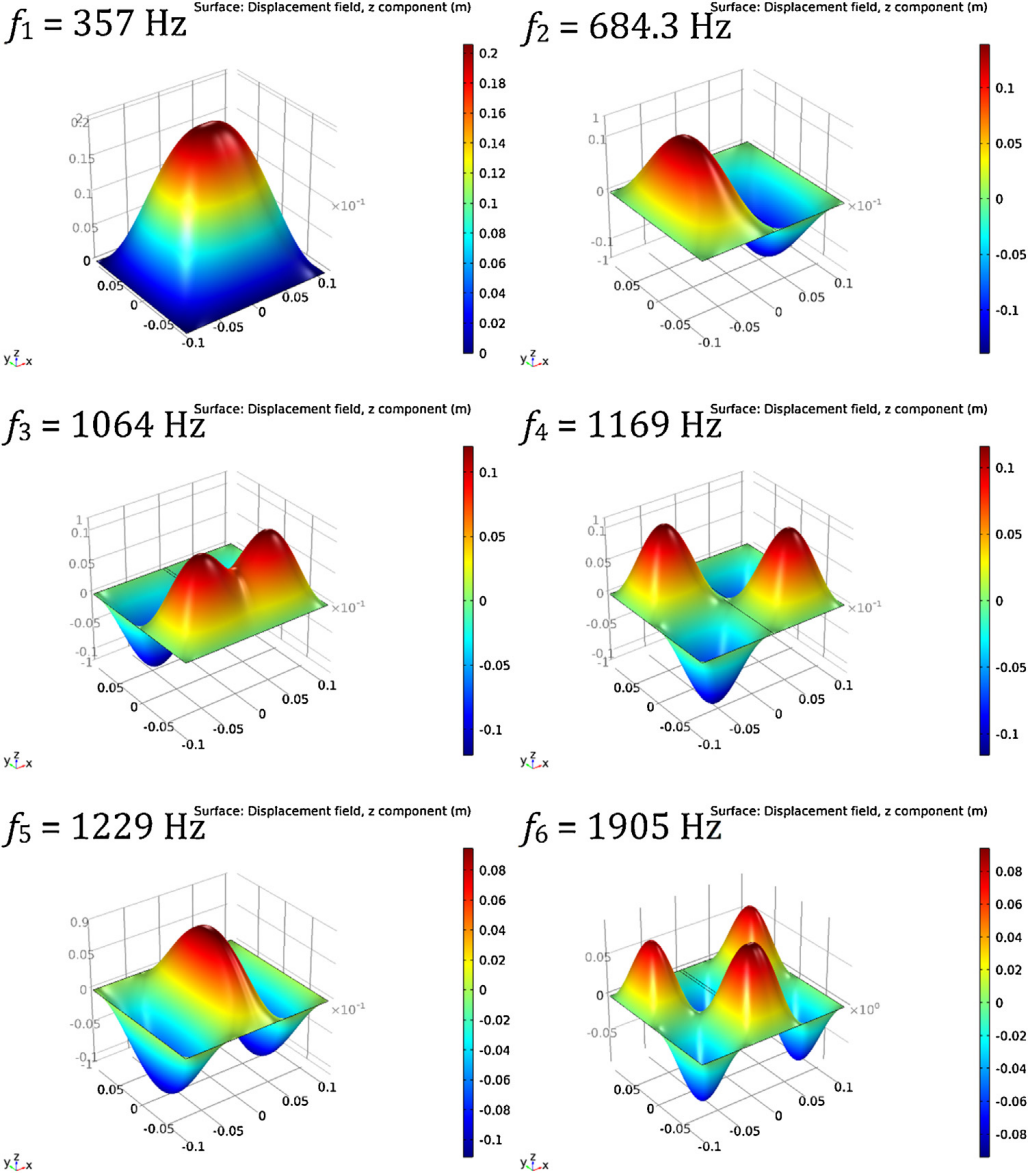
Results of the base ribbed plate FEA model for the lowest six modes of vibration.

**Fig. 16:**
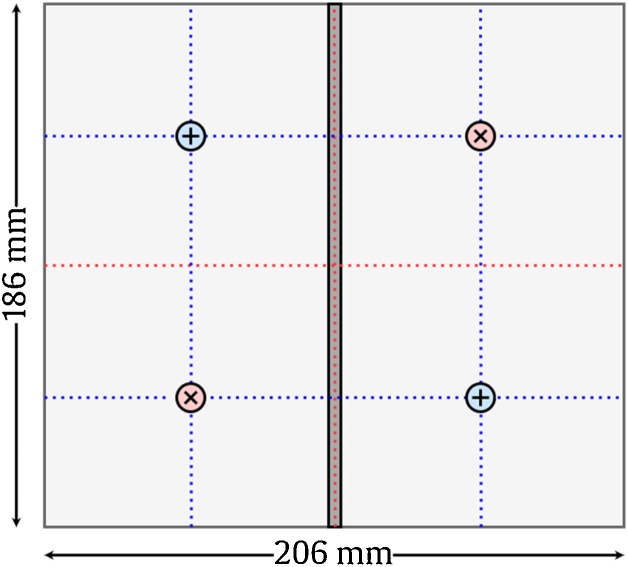
Location of accelerometers (+) and hammer impacts (x).

To capture the theoretical natural frequencies of the simply supported ribbed plate, a finite element analysis (FEA) model was created and simulated in COMSOL 5.1 using higher-order shear deformation theories. Results of the FEA model are given in Fig. 15. However, this model does not include the additional mass of the two accelerometers which would effectively lower the natural frequencies. Therefore, this model is referred to as the base model. A second augmented model was also created and simulated with accelerometer masses at their experimental location shown in Fig. 16.

A comparison of the results for both the base and augmented FEA models to their experimental counterparts is provided in Table 6. In this case, Base = theoretical frequencies for each mode calculated using the FEA model and Augmented = theoretical frequencies for each mode calculated using the FEA model with two additional accelerometer masses located as in Fig. 16. Sp/Ch and Im/Ac are the same as in Table 5.

**Table 6:** Theoretical and experimental natural frequencies of a simply supported ribbed rectangular plate.

Vibration modes of ribbed plate	Base (Hz)	Sp/Ch (Hz)	Error |%|	Augmented (Hz)	Im/Ac (Hz)	Error |%|
$m_{x}=1,\;m_{y}=1$	357.7	355	0.8	356.9	356.6	0.1
$m_{x}=2,\;m_{y}=1$	684.3	680	0.6	681.1	680.9	0.0
$m_{x}=1,\;m_{y}=2$	1,064	1062	0.2	1,056	1,056	0.0
$m_{x}=2,\;m_{y}=2$	1,169	1,156	1.1	1,158	1,158	0.0
$m_{x}=3,\;m_{y}=1$	1,229	-	-	1,226	1,226	0.0

For the ribbed plate, the average error when comparing experimental results to theoretical natural frequencies was 0.31% with a standard deviation of 0.39. In the case of the simple flat plates, the average error was 2.3% with a standard deviation of 1.72. In all cases, thicker plates provided better and more consistent results. This is likely due to the reduced impact that compressive stresses from mounting the plates in the supporting frame had on their material properties. Moreover, the largest errors occurred for the lowest frequency, likely due to the significant increase in resonance energy for this mode of vibration. Only a few outliers can be identified in the data and can likely be attributed to normal experimental and material variability. Overall, results confirm that the proposed setup provides reliable experimental measurements when compared to theory and should be used when multiple measurements must be taken relatively quickly or plates must be reused for other experiments or purposes.

### Additional information

The use of simply supported boundary conditions is generally favored in the theoretical modeling of vibrating plates due to their mathematical simplicity, as well as the availability of exact analytical solutions. Conversely, free and clamped boundary conditions are favored during experimental studies due to the ease in which they can be accurately represented experimentally. Unfortunately, the literature available for the experimental measurement of simply supported rectangular plates is sparse, as these are rather more difficult to model under real experimental constraints. One of the earliest attempts at experimentally modeling simply supported boundary conditions was performed by Hearmon on wooden plates [6]. In this study, a wooden frame was constructed using large v-grooves and long bolts which spanned the width of the plate in each direction. A similar method was developed by Amabili using sliding slots to accommodate various plate sizes and silicon to account for span shortening [7,8]. A similar experimental setup is discussed by Guo et al. [9,10] and used by Dumond and Baddour [11], the latter suggesting the use of removable sealant instead of silicon so that time between experimental trials is reduced and plate samples can easily be reused. Unfortunately, the edges of their plates had to be tapered to a point to properly sit in the v-grooves. The proposed method is based on the early work of Dumond and Baddour, providing the exact protocol for reproducing such experiments, but allowing plates to keep their original square edges and suggesting improvements, such as slots for surface mounting additional elements onto the plate. Alternatively, Barnard and Hambric propose that the supporting frame and the plate be made from one thick block of aluminum, where the plate is machined down in thickness and a groove is provided at its perimeter by only leaving a very thin webbing to support the plate [12]. While this approach is interesting, it does require that the plate and support be made of the same material and does not allow for span shortening. Alternatively, Hoppmann and Greenspon suggest clamping the edges of a larger plate and cutting grooves in the plate itself by notching to a depth of 80% of the total plate thickness at a perimeter line within the supported region which defines the actual plate dimensions of interest [13]. Obvious difficulties arise in using these plates for other purposes. Using a completely different approach, Ochs and Snowdon developed an experimental setup using a spring-steel skirt and support strip fixed to the plate using jewelers screws [14]. The spring-steel skirt is slotted to allow air to move freely around the vibrating plate and to allow for height adjustments of the setup. Pan et al. [15] and Yoon and Nelson [16] discuss similar setups. Champoux et al. provide guidelines and calculations for selecting the stiffness of the skirting material [17] which are based on the properties of the plate. Because of the difficulty inherent in using screws to fasten the edge of thin plates and the potential effects screw holes have on the results, Robin et al. suggest the use of a permanent adhesive instead [2]. However, the use of permanent adhesive limits the ability to use the plate following the vibration experiment. Moreover, a new spring-steel skirt must be defined and created for every plate used. In all cases, experimental results compare well to theoretical values and have been shown to be satisfactory. Although, the ease of experimental implementation varies greatly between methods. The method described herein provides a low-cost, simple, accurate and non-destructive way of experimentally measuring the modal properties of thin, simply supported plates and can be used for quick validations of models and designs without modification for multiple trials and varying plate properties.
